# Risk of Eye Damage from the Wavelength-Dependent Biologically Effective UVB Spectrum Irradiances

**DOI:** 10.1371/journal.pone.0052259

**Published:** 2012-12-20

**Authors:** Fang Wang, Qian Gao, Liwen Hu, Na Gao, Tiantian Ge, Jiaming Yu, Yang Liu

**Affiliations:** 1 School of Public Health, China Medical University, Shenyang, Liaoning, China; 2 Ophthalmology Department, The Fourth Affiliated Hospital of China Medical University, Shenyang, Liaoning, China; University of Tennessee, United States of America

## Abstract

A number of previous studies have discussed the risk of eye damage from broadband ultraviolet (UV) radiation. As the biologically damaging effectiveness of UV irradiation on the human body is known to be wavelength-dependent, it is necessary to study the distribution of the UV spectral irradiance. In order to quantify the ocular biologically effective UV (UVBE) irradiance exposure of different wavelengths and assess the risk of eye damage, UV exposure values were measured at Sanya, China (18.4° N, 109.7°E, altitude 18 m), using a manikin and a dual-detector spectrometer to measure simultaneously the ocular exposure and ambient UV spectral irradiance data and solar elevation angle (SEA) range (approximately 7°–85°). The present study uses the ocular UV spectral irradiance exposure weighted with the action spectra for photokeratitis, photoconjunctivitis and cataracts to calculate the ocular UVBE irradiance exposure for photokeratitis (UVBE_pker_), photoconjunctivitis (UVBE_pcon_) and cataracts (UVBE_cat_). We found that the ocular exposure to UV irradiance is strongest in the 30°–60° SEA range when ∼50% of ocular exposure to UV irradiance on a summer’s day is received. In the 7°–30° SEA range, all the biologically highly effective wavelengths of UVBE_pker_, UVBE_pcon_ and UVBE_cat_ irradiances are at 300 nm. However, in other SEA ranges the biologically highly effective wavelengths of UVBE_pker_, UVBE_pcon_ and UVBE_cat_ irradiances are different, corresponding to 311 nm, 300 nm and 307 nm, respectively.

## Introduction

Although solar ultraviolet (UV) radiation undergoes significant absorption by the atmosphere, both people and the environment will be exposed to higher intensity UV irradiance with depletion of the stratospheric ozone, with wide public health implications. In addition to skin cancer, it is impossible to ignore the risk of UV-related eye damage, which includes photokeratitis, photoconjunctivitis, cataracts, pterygium and age-related macular degeneration [1]–[3]. As solar UV radiation damage of the eye with increasing levels of UV irradiance is more detrimental than previously suspected [4], [5], people should become more aware of the risk from ocular exposure to solar UV radiation.

The links between solar UV radiation exposure and adverse ocular effects have been strengthened by animal experiments and epidemiological surveys [6]–[10]. However, quantitative measurement of ocular exposure to solar UV radiation is required in order to improve our understanding of the risk of eye damage from ocular exposure to solar UV radiation and so that appropriate strategies may be developed to measure ocular exposure to UV irradiance. Several previous studies have been conducted on the levels of ambient UV irradiance, measured horizontally, vertically or at different solar elevation angles (SEAs) [11]–[14].

As the eyeball is found in the orbit, whose structure is influenced by the facial anatomy [15], [16], a few studies have been attempted order to measure the dose of ocular exposure to UV irradiance using human subjects wearing instruments located at the side of eyeglasses [17]–[19]. Studies have also been conducted using manikins to simulate human ocular UV irradiance exposure [20]–[22].

Outdoors, ocular exposure to UV irradiance constantly changes during the day. Our previous study proposed a bimodal distribution of the diurnal variation in ocular exposure to solar UV waveband radiation, which is distinct from the horizontal ambient UV irradiance [20]. A bimodal distribution was also found by researchers who studied the diurnal variation of ocular exposure to solar UVA and UVB waveband radiation [21], [22].

The term action spectrum refers to the relative damaging effectiveness of UV irradiation in producing a particular biological response over a certain wavelength range [23], with different biological effectiveness corresponding to different action spectra [24]. The reliability and accuracy of any risk assessment or hazard evaluation of UV irradiance depend strongly upon the precision and accuracy of the relevant action spectra employed.

The biologically damaging effectiveness of solar UV radiation on human bodies is wavelength dependent and if one knows both the intensity and wavelength distribution of the UV irradiance one can combine any action spectrum with it and determine the biologically effective UV (UVBE) irradiance, which can quantitatively describe any biologically damaging effectiveness of solar UV radiation on human health. In order to obtain the ocular damaging UVBE spectrum irradiance, one should weight the solar UV spectral radiation that the eyes receive with the ocular damaging action spectrum. Spectroradiometers can provide the UV spectrum at 1 nm intervals, but only a few studies have applied the horizontal or vertical plane solar UV spectra irradiance weighted with the ocular damaging action spectra [25]–[27]. None of the previous studies measured ocular exposure to UV spectrum irradiance using manikins nor weighted it with the ocular damaging action spectra to provide information on the risk of eye damage from different wavelengths.

To assess accurately the risk of ocular damage due to exposure to solar UV irradiance, the current study measured the ocular exposure to UV spectrum irradiance using a spectroradiometer and a manikin, taking into account the characteristics of eye anatomical structure. The UV spectrum data was weighted by the photokeratital [28], photoconjunctivital [29] and cataractal [30] action spectra to calculate the ocular UVBE irradiance exposure. The diurnal variation of ocular UVBE irradiance exposure at different wavelengths of the UVB waveband at different SEAs, in addition to the biologically highly effective wavelengths for photokeratitis, photoconjunctivitis and cataracts, were determined in this study.

## Materials and Methods

### Experimental Instrument

**Figure 1:**
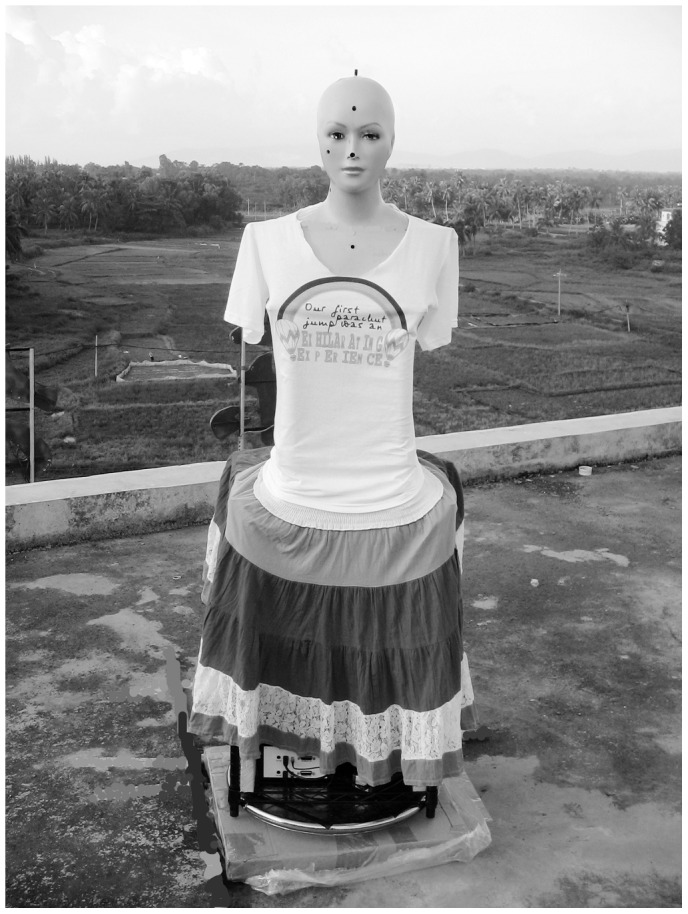
Manikin with two detectors monitoring ocular and ambient UV exposure.

The experimental instrument consisted of a turntable base, a middle shelf, and the upper part of a manikin (Fig. 1). The distance between eye and ground was ∼1.6 m. The interpupillary distance was ∼6 cm. The visual line was ∼10° below the horizon. The field of view of the manikin was ∼139°. The horizontal distance between the eye surface and the superciliary arch was ∼0.6 cm. The height of the nose bridge on the horizontal plane of the core of the eye was ∼0.6 cm. The UV intensity was measured using a computer-controlled spectrometer, which had two detectors and was placed on the shelf. One detector was on a plane tangent to the position of the right cornea at the most anterior point on the manikin and the other detector was placed at the vertex of the manikin head to ensure that the acceptance surface was horizontal to record simultaneously the ocular and ambient UV irradiance.

### Fiber Optic Spectrometer and Equipment Calibration

The fiber optic spectrometer and equipment calibration has been described by us previously [21].

### Geographical and Meteorological Conditions

Measurements were carried out in mid-July in the town of Hai Tang Wan near Sanya city (18.4° N, 109.7°E, 18 m above sea level), in the province of Hainan, China. Due to the fact that Sanya is the southernmost city in China, the maximum solar elevation angle (SEA) in mid-July is close to 90° and the average air temperature is 28.5°C. The site has a relatively unpolluted atmosphere with an average air pollution index (API) of 22 in 2010. In 2010, the percentage of days with superior air quality was 100% and the number of days with an API of best grade was 351.

The experimental instrument was placed ∼3 m and 1.5 m from the northern and the southern edges, respectively, of the asphalt-covered concrete roof of a five-story building that was surrounded by grassland. The five-story building was a hotel and the hotel owner called “Dingjun Xu” permitted us to carry out measurements on the roof of his hotel. The measurements were acquired on a sunny day with clear or only slightly cloudy skies. Measurement collection was determined by the weather forecast: we conducted measurements when the skies were clear, halted if cloud coverage occurred.

### UV Irradiance Measurements

Measurements of UV irradiance exposure were conducted on the 11th of July, 2010 from 08∶00 to 19∶00 China Standard Time (CST) (solar noon at about 12∶55 CST). The initial manikin position was such that the eye of the manikin pointed towards the sun every time. Each spectrometer detector collected data once per second, each measurement lasted 1 min and the measurement interval was 5 min. The manikin was rotated clockwise at a constant speed during data collection. The UV irradiance (unit µW cm^−2^ nm^−1^) at 1 s intervals was calculated over a range of 300–400 nm at 1 nm intervals. Overall, there were 60 groups of irradiance data per manikin revolution. The ocular maximum at different wavelength values of each revolution was calculated to simulate the actual maximum UV exposures under clear skies. The same procedure was carried out for data obtained from the ambient detector at the same time.

### UVBE Irradiance

**Figure 2:**
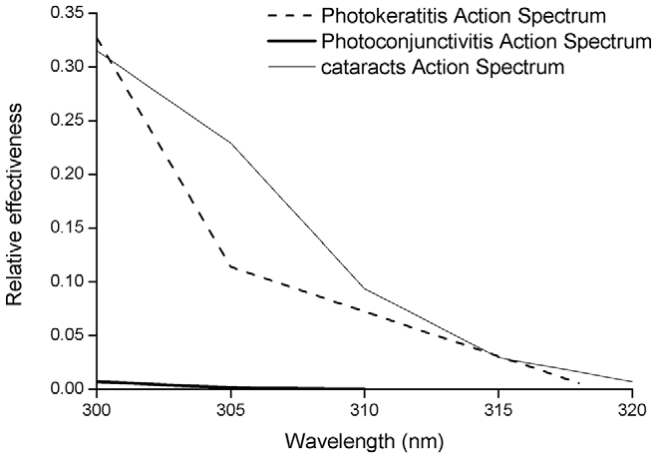
The photoconjunctivital, photokeratital and cataractal action spectra.

The UVBE irradiance is the spectral irradiance weighted with the action spectrum for a specific biological process, according to the following equation:
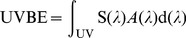
(1)where S(λ) is the measured spectral irradiance, A(λ) is the particular action spectrum and d(λ) is the wavelength increment of the spectral data, 1 nm in this case. For this study, the action spectra for photokeratitis [28], photoconjunctivitis [29] and cataracts [30] from 300 to 320 nm have been employed (Fig. 2). The relative effectiveness of the action spectrum decreases with increasing wavelengths for all biological processes. All the action spectra were linearly interpolated between the data points to 1 nm. In practice, the integral is substituted by a summation of the UV spectral irradiance with a certain wavelength range. In this work, we calculated both the UVBE of the wavelength increment at 1 nm and the integral summation from 300 to 320 nm.

### Exposure of UV Irradiance Dose

The UV irradiance dose (H) for specific time intervals was calculated as follows:
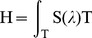
(2)where S(λ) is the measured spectral irradiance and T is the ocular exposure time interval. Again this was measured from 8∶08 CST (corresponding to a SEA of ∼25°) until 18∶42 CST (corresponding to a SEA of ∼7°). In this paper the SEA ranged from ∼7° to ∼85° with a maximum solar elevation of ∼85° at 12∶55 CST and we calculated the summation dosimetry of three different SEA ranges: from 7° to 30°, 30° to 60° and 60° to 85°, according to the relationship between the local time and the corresponding SEA. However, the dosimetry of the SEA range from 7° to 30° was partly missing in the morning as the SEA ranged from 7° to 25°. In order to ensure the integrity of this measured data, the morning data was added to that of the afternoon in the SEA range of 7° to 25°.

## Results

### Diurnal Variation for Selected Wavelengths

**Figure 3:**
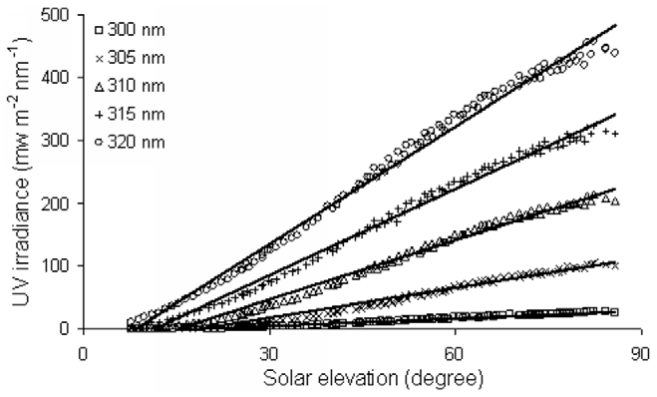
Ambient UVB irradiance of selected wavelengths at different SEAs and fitted regression curves.

**Figure 4:**
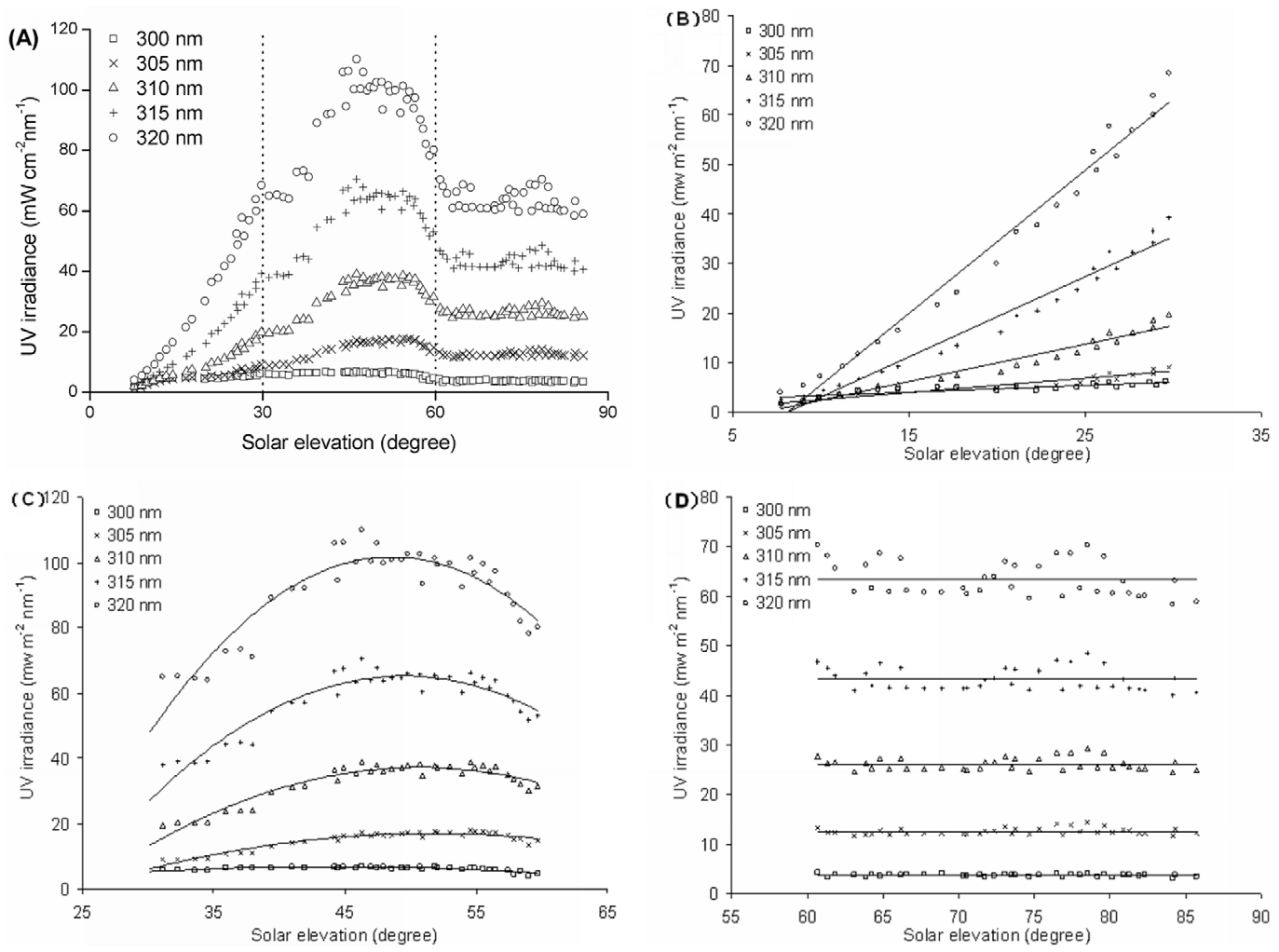
Ocular UVB irradiance of selected wavelengths at different SEAs (A) and fitted regression curves (B, C and D).

The diurnal variation of the ambient and ocular exposure to UV spectral irradiance for five selected wavelengths at different SEAs is shown in Figures 3 and 4A. The five selected wavelengths ranged from 300 nm to 320 nm at 5 nm intervals. The ambient UV irradiance intensity increased with increasing solar elevation at all selected wavelengths with the highest ambient UV irradiance measured at the highest solar elevation. At the same SEA, UV irradiance intensity increased with increasing wavelength (Fig. 3). The fitted equations of the fitted regression curves for ambient UV irradiance are shown in Table 1 and fit the linear function in the 7°–85° SEA range.

**Table 1:** Fitted equations of ambient and ocular exposure to UVB irradiance at the selected wavelengths.

Wavelength	Ambient (7°–85°)		Ocular (7°–30°)		Ocular (30°–60°)		Ocular (60°–85°)
	Fitted equations	R^2^	Fitted equations	R^2^	Fitted equations	R^2^	Fitted equations
**300 nm**	y = 0.4003x –7.049	R^2^ = 0.97	y = 0.1369x +1.8933	R^2^ = 0.73	y = −0.0079x^2^+0.6932x−8.5774	R^2^ = 0.74	y = 3.65
**305 nm**	y = 1.569x –29.468	R^2^ = 0.98	y = 0.2864x−0.3743	R^2^ = 0.92	y = –0.0236x^2^+2.4297x−45.79	R^2^ = 0.91	y = 12.51
**310 nm**	y = 3.1503x –47.894	R^2^ = 0.99	y = 0.7509x−5.0743	R^2^ = 0.95	y = –0.0565x^2^+5.725x−108.02	R^2^ = 0.91	y = 26.12
**315 nm**	y = 4.59x –52.882	R^2^ = 0.99	y = 1.6218x−13.211	R^2^ = 0.97	y = –0.1015x^2^+10.047x−183.48	R^2^ = 0.88	y = 43.21
**320 nm**	y = 6.246x –53.179	R^2^ = 0.99	y = 2.8894x−23.372	R^2^ = 0.98	y = –0.1584x^2^+15.393x−272.18	R^2^ = 0.86	y = 63.47

The diurnal distribution of ocular exposure to UV irradiance at different SEAs is markedly different from that of the ambient (Fig. 4A). In order to describe the diurnal variation characteristics of ocular exposure to UV irradiance at different solar elevations, this paper divides the total SEA range into three. In the 7°–30° SEA range, the distribution of the selected wavelengths of ocular exposure to UVB irradiance all increased with increasing solar elevation, as did ambient UV irradiance in the same SEA range (Figs. 3, 4B). However, in the 30°–60° SEA range, the distribution of the selected wavelengths of ocular exposure to UVB irradiance are similar to bell-shaped curves, with peaks at ∼50° SEA (Fig. 4C), while in the 60°–85° SEA range, the distribution of the selected wavelengths of ocular exposure to UVB irradiance shows a distribution that is largely parallel with the x-axis (Fig. 4D).

At the same SEA, ocular exposure to UV irradiance intensity increased with higher wavelengths. The fitted equations of the fitted regression curves for ocular exposure to UV irradiance fit the linear function, quadratic function and constant function, which is parallel to the x-axis, corresponding to the SEA ranges from 7° to 30°, 30° to 60° and 60° to 85°, respectively, and are shown in Table 1.

### Dosimetry Ratios for Selected Wavelengths

**Figure 5:**
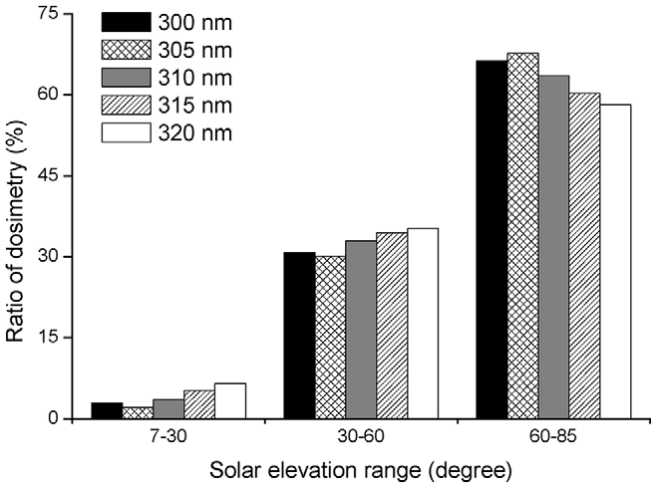
Ratio of ambient UVB irradiance of three SEA ranges to total SEA for selected wavelengths.

The dosimetry ratio for a wavelength is calculated from the dose at a particular SEA range for this wavelength to the dose of the total SEA range at the same wavelength. The percentage dosimetry ratios of the selected wavelengths of ambient UV irradiance were 2–7%, 30–35%, 58–68%, corresponding to the SEA ranges of 7°–30°, 30°–60° and 60°–85°, respectively (Fig. 5). The dosimetry ratios of the selected ambient UV irradiance wavelengths in the 60°–85° SEA range were between 9 times greater (at 320 nm) and 32 times greater (at 305 nm) than the dosimetry ratios of the corresponding wavelengths in the 7°–30° SEA range.

**Figure 6:**
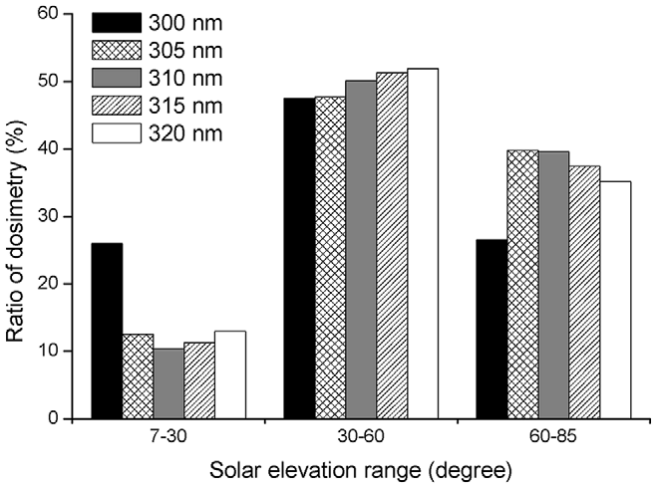
Ratio of ocular UVB irradiance of three SEA ranges to total SEA for selected wavelengths.

For ocular exposure to UV irradiance, the dosimetry ratio at 300 nm was 26%, which was markedly higher than the 13–16% seen for the ratios of the other selected wavelengths in the 7°–30° SEA range. However, the dosimetry ratio was only 26.5% in the 60°–85° SEA range, which was markedly lower (between 8.5 and 13.5%) than the ratio of the other selected wavelengths at the same SEAs. The dosimetry ratios of the other selected wavelengths were 10–13% and 35–40% in the 7°–30° and 60°–85° SEA ranges, respectively. The ratios of the selected wavelengths were all about 50% in the 30°–60° SEA range (Fig. 6). Additionally, except for the dosimetry ratio of ocular exposure to UV irradiance at 300 nm in the 60°–85° SEA range, which was similar to the dosimetry ratio in the 7°–30° SEA range, the dosimetry ratios of ocular exposure to UV irradiance in the 60°–85° SEA range of the other selected wavelengths were about three times greater than the dosimetry ratios in the 7°–30° SEA range.

### Diurnal Variation of UVBE Irradiance

**Figure 7:**
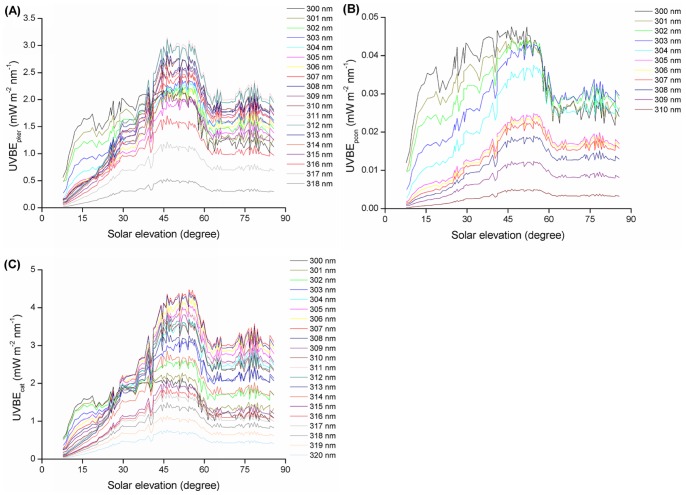
Ocular UVBE irradiances at different SEAs: (A) UVBE_pker_, (B) UVBE_pcon_, (C) UVBE_cat_.

Limited by the action spectra, the maximum wavelengths of the UVBE irradiance for photokeratitis (UVBE_pker_) and photoconjunctivitis (UVBE_pcon_) end at 318 nm and 310 nm, respectively (Figs. 7A, B). As this paper focuses on the biologically damaging effectiveness of the UVB waveband, the maximum wavelength of the UVBE irradiance for cataracts (UVBE_cat_) ends at 320 nm. The maximum intensities of UVBE_pker_, UVBE_pcon,_ and UVBE_cat_ irradiance were 3.15, 0.05 and 4.5 mW m^−2 ^nm^−1^, respectively and were found at a SEA of ∼50°. At the same SEA the intensity of UVBE_pcon_ irradiance was decreased for the higher wavelengths in all SEA ranges (Fig. 7B), whereas the intensity distribution of UVBE_pker_ and UVBE_cat_ irradiance for different wavelengths in the same SEA range were different from that of UVBE_pcon_ irradiance. At the same SEA the intensity of UVBE_pker_ and UVBE_cat_ irradiance decreased with increasing wavelength in the 7°–30° SEA range, but no regular pattern was seen in the SEA range of 30°–85°. However, the wavelengths of maximum intensity were seen at the same SEA at 311 nm and 307 nm for UVBE_pker_ and UVBE_cat_, respectively (Figs. 7A, C).

### Dosimetry of UVBE Irradiance

**Figure 8:**
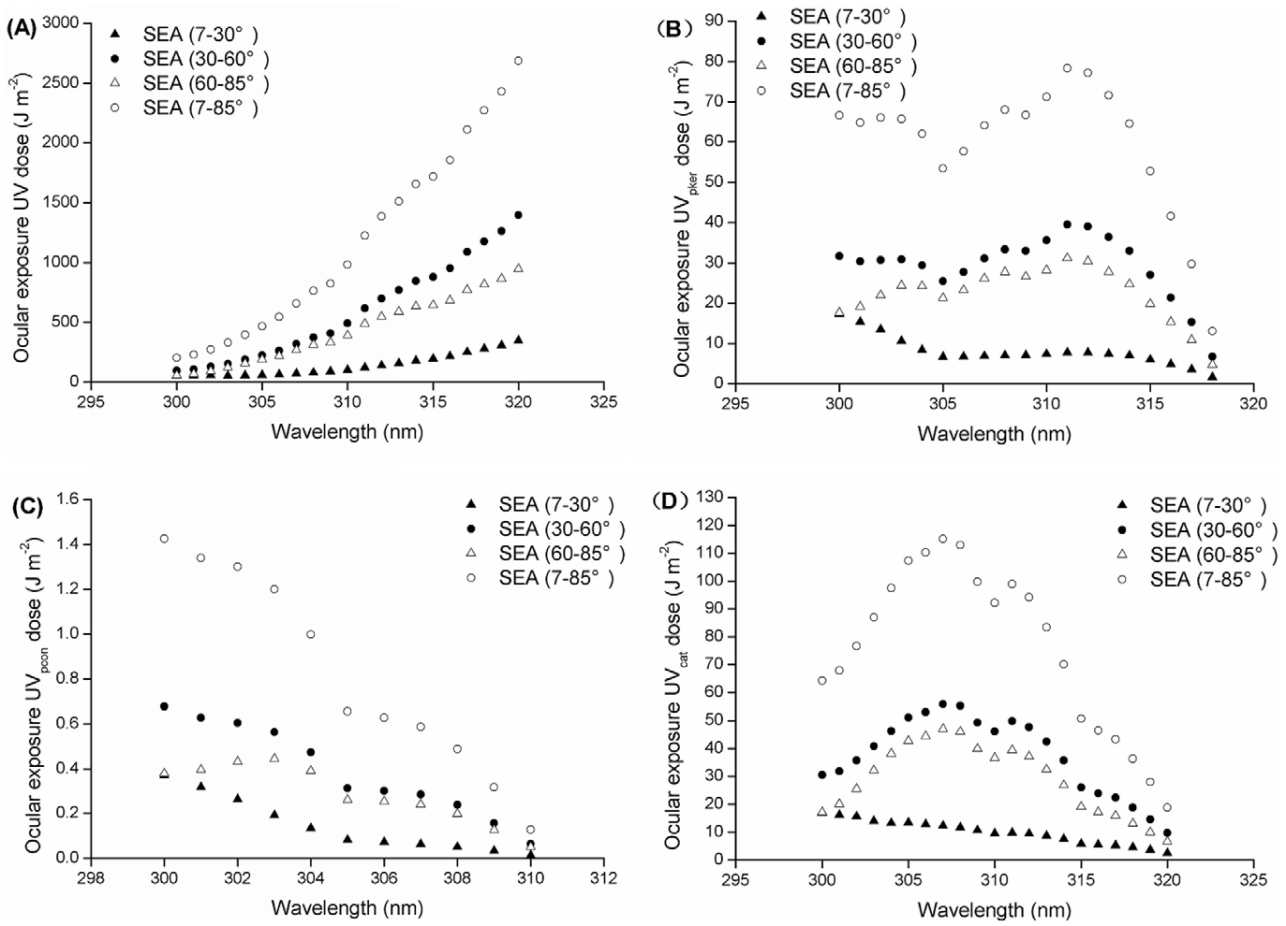
Dosimetry of UV spectrum irradiance: (A) ocular UV, (B) UVBE_pker_, (C) UVBE_pcon_, (D) UVBE_cat_.

The dosimetry distribution of ocular UV irradiance exposure shows that the dose increases in line with the wavelength in three SEA ranges (7°–30°, 30°–60° and 60°–85°) as well as in the total (7°–85°) SEA range (Fig. 8A), while the distribution of the UVBE_pcon_ irradiance dosimetry was contrary to the ocular exposure to UV irradiance (Fig. 8C). Furthermore, it is clear that there is not a simple dosimetry distribution for UVBE_pker_ and UVBE_cat_ irradiance. The dose decreases with increasing wavelength in the 7°–30° SEA range for UVBE_pker_ and UVBE_cat_ (Figs. 8B, D) but in the 30°–60°, 60°–85° and 7°–85° SEA ranges, the dosimetry distribution of UVBE_pker_ shows a peak at 311 nm and a valley at 305 nm (Fig. 8B), while the dosimetry distribution of UVBE_cat_ shows a similar bell-shaped curve with a peak of 307 nm (Fig. 8D).

Wavelengths close to the strongest biologically effective wavelength have a relatively strong effect. The highly effective wavebands for UVBE_pker,_ UVBE_pcon_ and UVBE_cat_ in the total SEA range are from 309 to 313 nm, 300 to 304 nm and 305 to 309 nm, respectively. Additionally, at the same wavelength, the dosimetry of the 30°–60° SEA range was clearly higher than that of the 7°–30° SEA range and slightly higher than that of the 60°–85° SEA range.

### Dosimetry Ratio for the Summed UVB

Table 2 shows that the maximum and the minimum ratios of ambient UVB waveband irradiance were in the 60°–85° and 7°–30° SEA ranges, respectively. The maximum value was about 13 times the minimum. However, for ocular exposure and ocular biologically effective UVB waveband radiation exposure, the maximum and the minimum ratios were in the 30°–60° and 7°–30° SEA ranges and the maximum value was only about 3 to 4 times the minimum. Additionally, the ratio of ocular exposure to the biologically effective UVB waveband irradiance was higher than the ratio of ocular exposure to the UVB waveband irradiance. In the 7°–30° SEA range the dosimetry ratio of UVBE_pcon_ was higher than that of UVBE_pker_ and UVBE_cat_.

**Table 2:** Ratio of the summed UVB exposure dosimetry of three SEA ranges to total SEA range.

SEArange	UVBE_pker_	UVBE_pcon_	UVBE_cat_	Ocular UVexposure	Ambient UV
**7°–30°**	13.06	16.87	12.72	11.8	4.78
**30°–60°**	49.16	47.49	49.16	50.64	33.75
**60°–85°**	37.78	35.65	38.12	37.56	61.47
**7°–85°**	100	100	100	100	100

## Discussion

In this study, measurements were simultaneously performed with a head manikin in order to obtain ocular exposure and ambient UV spectrum irradiance data. Five selected wavelengths of the UVB waveband were used to investigate the diurnal variations of ocular exposure and ambient UV irradiances.

We found that the diurnal variations of ocular exposure to UV irradiances have their own characteristics in the 7°–30°, 30°–60° and 60°–85° SEA ranges. In order to show these characteristics we separated the distribution of ocular UV irradiance exposure into three SEA ranges and obtained the fitted equations that corresponded to each part. The fitted equations in the 7°–30°, 30°–60° and 60°–85° SEA range fit the linear function, quadratic function and the constant function (parallel to the x-axis), respectively. The diurnal distribution of UV ocular exposure is different from the distribution of the horizontal ambient UVB irradiances where all the fitted equations fit the linear function in the total SEA range.

This paper calculates the dosimetry ratios of ambient and ocular exposure to UVB spectrum irradiances of three SEA ranges to the total SEA range at specific wavelengths. For the ratio of ambient UV spectrum irradiance at these wavelengths, we found that the maximum ratio of the wavelengths in the three different SEA ranges of ambient UV irradiance reached ∼60% in the 60°–85° SEA range around noon when the UV rays are strongest. The maximum ratio of the selected wavelengths in the SEA range of 60°–85° is several times greater than the minimum ratio in the SEA range of 7°–30° for the same wavelength. However, for the ratio of ocular exposure to UV spectrum irradiance of the wavelengths in the three different SEA ranges to the total SEA range, we found that the maximum ratio of ocular exposure to the UV spectrum irradiance was ∼50% of all the selected wavelengths in the 30°–60° SEA range. Thus the dosimetry distribution of the ambient and ocular exposure to UVB spectrum irradiance varies considerably.

This work suggests that the high-risk period for eye-damaging solar UV radiation is not only at noon but also in the morning and afternoon in the 30°–60° SEA range. Additionally, the ratio of ocular exposure to UV irradiance in the 60°–85° SEA range at the 300 nm wavelength is similar to the ratio of ocular exposure to UV irradiance in the 7°–30° SEA range. However, the ratio of ocular exposure to UV irradiance in the 60°–85° SEA range of the other selected wavelengths is about three times greater than the ratio of ocular exposure to UV irradiance in the 7°–30° SEA range. As the ratio of diffuse to direct UV irradiances decreases rapidly with increasing wavelength at the same air composition (gaseous pollutants, aerosols, etc.) and solar zenith angle (SZA) [31], this paper suggests that the particularity of the 300 nm wavelength, where the ratio is markedly higher than other selected wavelengths in the 7°–30° SEA range, may be associated with a greater effect of scattering and diffusion on shorter wavelengths at low SEAs.

The biologically damaging effectiveness of UV irradiances on human bodies is dependent on the UVBE irradiance intensity. The horizontal ambient UVBEpker and UVBEcat irradiances were investigated by Parisi and Downs [25], who measured the horizontal ambient UV irradiances on an unshaded roof at a sub-tropical latitude in Toowoomba (27.5° S, 152.0°E, 693 m above sea level), Australia. The UVBEpker and UVBEcat irradiance figures at cloud-free periods for 6°, 51° and 71° solar zenith angles (SZAs) showed that the UVBEpker and UVBEcat irradiances increased with a smaller SZA and the maximum UVBEpker and UVBEcat irradiances were ∼20 mW m^−2^ nm^−1^ and ∼30 mW m^−2^ nm^−1^, respectively. However, the present study, which investigated the UVBE_pker_, UVBE_pcon_ and UVBE_cat_ spectrum irradiances reaching human eyes, found that the maximum value of ocular UVBEpker and UVBEcat irradiances occurred at a SEA of ∼50° and were 3.15 mW m^−2^ nm^−1^ and 4.5 mW m^−2^ nm^−1^, respectively. These disparate results can be explained by the differences between the two studies. For example, in the study of Parisi and Downs, the measured location was 693 m above sea level, while in the present study the elevation was 18 m; the increase of UV irradiances per 1000 m altitude only confers a 20% at 320 nm, and increases to 30% at 300 nm in sunny weather [32].

Furthermore, Parisi and Downs measured UV irradiance on an unshaded roof and did not mention the surface material. In this paper the solar UV radiation was measured on a roofing surface covered with asphalt; this distinction is important as the measured UV irradiance is influenced by ground reflectance factors [33]. Tanskanen and Manninen consider that at ultraviolet wavelengths the albedo of most surfaces is small, with the exception of snow and ice [34]. In this study, the maximum horizontal ambient UVBEpker and UVBEcat irradiances that were calculated with the horizontal ambient UV irradiance weighted with the action spectra for photokeratitis and cataracts at 84° SEA were 16 mW m^−2^ nm^−1^ and 25 mW m^−2^ nm^−1^, respectively, similar to the results of Parisi and Downs. Moreover, we consider the fact that horizontal ambient UV irradiance data was used in the Parisi and Downs study while ocular exposure to UV irradiances, measured using a manikin, was used in this study to be the most important reason for the differing results.

In this study we found that the diurnal distribution of ocular UVBE_pker_, UVBE_pcon_ and UVBE_cat_ irradiance exposure for different wavelengths at different SEAs were different from the ocular exposure to UV irradiances, which increases with larger wavelengths at the same SEA. In the total (7°−85°) SEA range, the maximum ocular UVBE_pcon_ irradiance exposure intensity of different wavelengths at the same SEA was at 300 nm. In the 7°−30° SEA range, the maximum ocular UVBE_pker_ and UVBE_cat_ irradiance exposure intensity of different wavelengths at the same SEA were both at 300 nm. However, in the 30°−85° SEA range, the maximum ocular UVBE_pker_ and UVBE_cat_ irradiance exposure intensity of different wavelengths at the same SEA was at 311 nm and 307 nm, respectively. Additionally, in the total SEA range the highly effective waveband for ocular exposure to UV irradiance is from 316 to 320 nm. However, the highly effective wavebands for ocular UVBE_pker,_ UVBE_pcon_ and UVBE_cat_ exposure are from 309 to 313 nm, 300 to 304 nm and 305 to 309 nm, respectively, according to the dosimetry distribution of UVBE irradiance at 1 nm interval wavelengths.

From the dosimetry ratio of the summed UVB waveband exposure in the three different SEA ranges to the exposure in the total SEA range, we found that the ratio of the ocular biologically effective UVB waveband irradiance exposure is higher than the ratio of ocular UVB waveband irradiance exposure and the ratio of ocular UVBE_pcon_ exposure is higher than the ratio of ocular UVBE_pker_ and UVBE_cat_ exposure in the 7°−30° SEA range. The above results show that the biologically effectiveness of UV spectrum irradiance on ocular damage is different at different wavelengths and that the highly effective UV spectral waveband for photokeratitis, photoconjunctivitis and cataracts is different.

As the UVBE irradiance was calculated with the UV spectrum irradiance weighted by the relative action spectrum, the above data is dependent on the intensity of the UV spectrum irradiance and the action spectra. As long as the measured data of ocular exposure influences UV spectrum irradiances at different wavelengths, the ocular exposure UVBE irradiances will also change. This research was performed on a sunny day. The influence of cloud-layer and air pollution on UV radiances is wavelength-dependent [35], [36]. Accordingly, further research should be undertaken under different weather conditions, especially black cloud or serious air pollution, as they are likely to have different results.

This paper shows the diurnal distribution at SEAs ranging from 7° to 85° and their contribution to ocular damage at different wavelengths with normal maximum UV exposure in clear sky conditions. This study proves that it can be assumed that the UVBE_pker_, UVBE_pcon_ and UVBE_cat_ irradiances are wavelength-dependent. Additionally, the risk assessment of eye damage from the UVBE_pker_, UVBE_pcon_ and UVBE_cat_ irradiances need comprehensive evaluation with regard to the SEA range and the intensity of UV irradiance of different wavelengths.
